# The Influence of Hydrogen Bond Donors on the CO_2_ Absorption Mechanism by the Bio-Phenol-Based Deep Eutectic Solvents

**DOI:** 10.3390/molecules26237167

**Published:** 2021-11-26

**Authors:** Ze Wang, Zonghua Wang, Jie Chen, Congyi Wu, Dezhong Yang

**Affiliations:** School of Science, China University of Geosciences, Beijing 100083, China; wangze666wz@163.com (Z.W.); wzh13263359399@126.com (Z.W.); chenjie@cugb.edu.cn (J.C.); wucongyi@cugb.edu.cn (C.W.)

**Keywords:** capture, carbon dioxide, deep eutectic solvents, insights, spectroscopy

## Abstract

Recently, deep eutectic solvents (DESs), a new type of solvent, have been studied widely for CO_2_ capture. In this work, the anion-functionalized deep eutectic solvents composed of phenol-based ionic liquids (ILs) and hydrogen bond donors (HBDs) ethylene glycol (EG) or 4-methylimidazole (4CH_3_-Im) were synthesized for CO_2_ capture. The phenol-based ILs used in this study were prepared from bio-derived phenols carvacrol (Car) and thymol (Thy). The CO_2_ absorption capacities of the DESs were determined. The absorption mechanisms by the DESs were also studied using nuclear magnetic resonance (NMR), Fourier transform infrared (FTIR), and mass spectroscopy. Interestingly, the results indicated that CO_2_ reacted with both the phenolic anions and EG, generating the phenol-based carbonates and the EG-based carbonates, when CO_2_ interacted with the DESs formed by the ILs and EG. However, CO_2_ only reacted with the phenolic anions when the DESs formed by the ILs and 4CH_3_-Im. The results indicated that the HBDs impacted greatly on the CO_2_ absorption mechanism, suggesting the mechanism can be tuned by changing the HBDs, and the different reaction pathways may be due to the steric hinderance differences of the functional groups of the HBDs.

## 1. Introduction

The climate crisis has been one of the most serious issues threatening the environment, animals, and humankind, which is regarded as the feedback of global warming. Greenhouse gases generated from human activity are the major cause of climate change, which mainly include fluorinated gases, methane (CH_4),_ carbon dioxide (CO_2_), and nitrous oxide (N_2_O) [1]. Among these gases, CO_2_ is the main contributor, mainly released from the combustion process of fossil fuels (e.g., crude oil and coal) in the industrial sectors, such as power plants, cement kilns, and ammonia production [2]. With the rapid development of the global economy in recent decades, resulting in the huge combustion of fossil fuels, the atmospheric CO_2_ concentration has reached more than 410 ppm in 2020 [3]. In order to reduce and control the CO_2_ emission into the atmosphere, many technologies have been developed and used in industry, of which chemical absorption processes based on amine aqueous solutions are the most used methods because of the relatively high capture efficiency [4,5]. However, the amine-based absorption technology suffers inherent shortcomings, including the high energy cost of the regeneration processes, serious equipment corrosions, and the high volatility of the solvents [6].

In recent decades, ionic liquids (ILs) have been widely investigated to capture CO_2_ because of their unique properties, such as high thermal stability, tunable structures, and low vapor pressure [7]. Of them, functional ILs, including amine-based ILs, azolide-based ILs, and phenol-based ILs, which could chemically capture CO_2_ due to the active sites on the cations or anions, exhibited high capacities for CO_2_, making them promising candidates as chemical solvents to capture carbon [8]. However, the complex synthesis procedures and the high cost are the main obstacles limiting their practical applications.

Deep eutectic solvents (DESs), exhibiting similar properties of ILs, have gained great attentions in recent years and they are also studied as solvents for CO_2_ absorption [9,10]. At present, DESs are mainly formed by mixing hydrogen bond donors (HBDs) with hydrogen bond acceptors (HBAs) through the intermolecular hydrogen bonding interactions between HBDs and HBAs, which results in the formation of liquid solvents with lower melting points than the individual components in the system [11]. A lot of DESs have been prepared as CO_2_ absorbents so far. Among them, amine-based DESs [12,13,14,15,16], anion-functionalized DESs [17,18], and superbase-based DESs [19,20,21,22] showed attractive CO_2_ capacities through the reaction between CO_2_ and active sites in the components at ambient pressure, suggesting that adjusting the structures of the components in the DESs can be an effective strategy to meet the particular demands. Moreover, the interactions between CO_2_ and the functionalized DESs were also reported in the literatures. In our previous work, we found that CO_2_ reacted with ethylene glycol when CO_2_ was captured by the DESs composed of azolide-based ILs and EG, but CO_2_ did not react with the azolide anions in the DESs [17]. However, CO_2_ reacted with both the azolide anions and EG in the DESs formed by the superbase-derived ILs and EG [20]. Another different reaction pathway between CO_2_ and DESs was reported when CO_2_ interacted with the DESs composed of polyamine-based ILs and EG, in which CO_2_ reacted with the amine groups on the cations and did not react with the [Im]^−^ anion of the ILs in the solvent [18]. These results suggest that it is important to know the roles that the components of DESs take part in when DESs interact with CO_2_, which will be useful to the design of new DESs for efficient CO_2_ absorption.

**Scheme 1:**
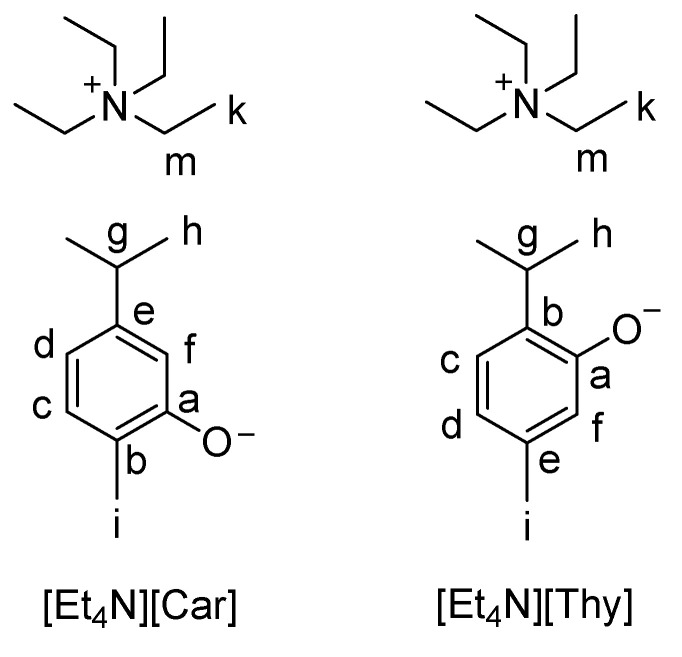
The structures of the phenol-based ILs, [Et_4_N] [Car] and [Et_4_N] [Thy]. Letters from a to i are the labels of hydrogen or carbon atoms of [Car] or [Thy] anion. Letters m and k are the labels of hydrogen or carbon atoms of [Et_4_N] cation.

In this work, we report that the CO_2_ absorption mechanism by DESs based on phenol-derived ILs can be tuned by changing the HBDs. The DESs used in this work were prepared by mixing phenol-based ILs with HBDs ethylene glycol (EG) or 4-methylimidazole (4CH_3_-Im). The phenol-based ionic liquids used in this study can be easily synthesized by the acid–base reactions between tetraethylammonium hydroxide ([Et_4_N][OH]) aqueous solution and the bioderived phenols, including carvacrol (Car) and thymol (Thy). The obtained ILs [Et_4_N] [Car] and [Et_4_N] [Thy] are solids at room temperature (Scheme 1). After they were mixed with the solid 4CH_3_-Im at a 1:2 molar ratio, the liquid solvents [Et_4_N] [Car]:4CH_3_-Im (1:2) and [Et_4_N] [Thy]:4CH_3_-Im (1:2) can be obtained. Similarly, DESs [Et_4_N] [Car]:EG (1:2) and [Et_4_N] [Thy]:EG (1:2) can also be obtained by mixing the solid ILs with EG. The absorption mechanism by these DESs was investigated, and the results indicated that the reaction pathway of CO_2_ with EG-based DESs was different from that of CO_2_ with 4CH_3_-Im-based DESs, suggesting the important role of HBDs in the DESs for CO_2_ capture.

## 2. Results and Discussion

**Figure 1:**
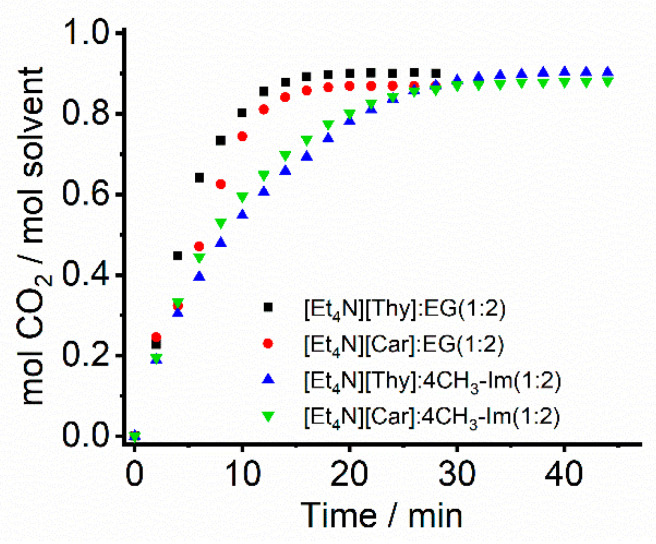
The CO_2_ absorption by the DESs [Et_4_N] [Thy]:EG (1:2), [Et_4_N] [Car]:EG (1:2), [Et_4_N][Thy]:4CH_3_-Im (1:2), and [Et_4_N] [Car]:4CH_3_-Im (1:2) at 25 °C and 1.0 atm.

The melting point (*T*_m_) and decomposition temperature (*T*_d_) of the DESs used in this work were investigated, and the results were shown in Table S1. The melting points of EG-based DESs were not observed in the temperature range studied (−80–25°C). The viscosities of the four DESs were determined at 25 °C (Table S1). The CO_2_ absorption capacities of the four phenol-based DESs were measured at 1.0 atm and 25 °C (Figure 1). The DESs [Et_4_N] [Thy]:EG (1:2), [Et_4_N] [Car]:EG (1:2) [Et_4_N] [Thy]:4CH_3_-Im (1:2), and [Et_4_N] [Car]:4CH_3_-Im (1:2) could capture 0.90, 0.87, 0.90, and 0.88 mol CO_2_/mol solvent, respectively. These results indicated that these four phenol-based DESs can efficiently capture CO_2_. Moreover, as shown in Figure 1, CO_2_ was faster to reach saturation in EG-based DESs than in 4CH_3_-Im-based DESs. This phenomenon was mainly due to the influence of the viscosity of the solvent. The viscosities of [Et_4_N][Car]:EG (1:2) and [Et_4_N][Thy]:EG (1:2) were 279 and 292 mPa·s at 25 °C, respectively, which were much lower than those of [Et_4_N][Car]:4CH_3_-Im (1:2) (891 mPa·s) and [Et_4_N][Thy]:4CH_3_-Im (1:2) (1298 mPa·s). The low viscosity of EG-based DESs was beneficial to CO_2_ diffusion in the solvent, which may promote the reactions between CO_2_ and DESs, thus EG-based DESs exhibited a faster CO_2_ absorption rate. The comparison of CO_2_ capacities by DESs used in this work with other DESs previously reported was shown in Table S2.

The effect of temperature on CO_2_ capacity of the [Et_4_N][Car]:4CH_3_-Im (1:2) and [Et_4_N][Car]:EG (1:2) DESs was studied (Figure S1). As shown in Figure S1, the CO_2_ capacity decreased with increasing temperature. The CO_2_ desorption by the DESs used in this work was also investigated. The captured CO_2_ by DESs can be released at 80 °C. As an example, the reversibility of [Et_4_N][Car]:EG(1:2) was studied. The five consecutive absorption–desorption cycles of [Et_4_N][Car]:EG(1:2) were shown in Figure S2, and the result suggested [Et_4_N][Car]:EG(1:2) can be reused.

**Figure 2:**
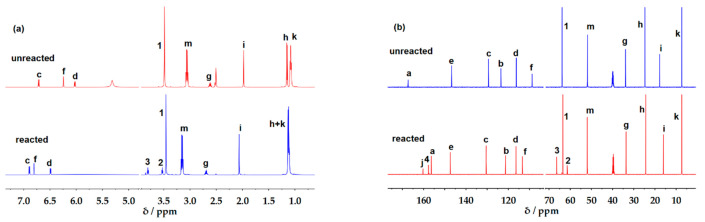
The ^1^H (**a**) and ^13^C (**b**) NMR spectra of [Et_4_N] [Car]:EG (1:2) before and after CO_2_ uptake. Letters from a to i are the labels of hydrogen or carbon atoms of [Car] anion with and without CO_2_. Letters m and k are the labels of hydrogen or carbon atoms of [Et_4_N] cation. Letter j is the label of carbonyl carbon of Car-based carbonate. Numbers 1 is the label of hydrogen or carbon atom of EG. Numbers 2, 3 and 4 are the labels of hydrogen or carbon atoms of EG-based carbonate.

In order to clarify the absorption mechanism, nuclear magnetic resonance (NMR) spectroscopy and Fourier transform infrared (FTIR) spectroscopy were used to study the reaction between CO_2_ and the DESs. The NMR spectra of [Et_4_N] [Car]:EG (1:2) before and after CO_2_ uptake were shown in Figure 2. As seen in the ^1^H NMR spectra, two new peaks at 3.53 (H-2) and 3.80 (H-3) ppm can be observed after CO_2_ absorption. In the ^13^C NMR spectra, four new peaks at 61.4 (C-2), 66.4 (C-3), 157.7 (C-4), and 160.3 (C-j) ppm can be found after absorption. Moreover, H-f and H-d shifted downfield from 6.24 and 6.02 ppm to 6.80 and 6.48 ppm, respectively. C-a shifted upfield from 167.3 to 156.3 ppm and C-f shifted downfield from 108.8 to 113.4 ppm. It is reasonable to expect that CO_2_ could react with the anion [Car]^−^ in the solvent because of the acid–base interactions, which would produce a carbonate species, and then a new carbon signal of the carbonate carbonyl carbon would be found in the ^13^C NMR spectra. However, if CO_2_ only reacts with the anion, it is difficult to explain why there are four new peaks in the ^13^C NMR spectra after absorption. As reported in our previous work [17], CO_2_ reacted with the -OH group of EG, forming a carbonate species when it was captured by the DESs composed of azolide ILs and EG. Therefore, we think that CO_2_ may also react with EG in the DESs [Et_4_N] [Car]:EG (1:2).

**Figure 3:**
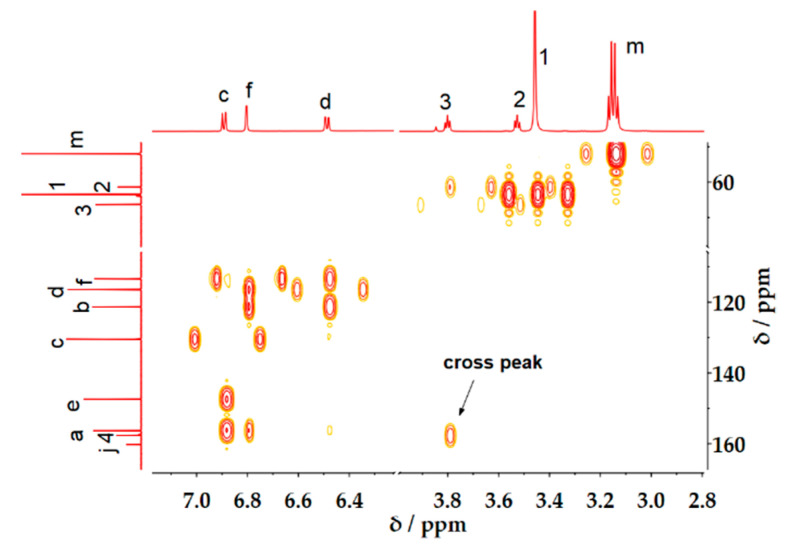
The ^1^H–^13^C HMBC spectrum of [Et_4_N] [Car]:EG (1:2) + CO_2_. Letters from a to j are the labels of hydrogen or carbon atoms of Car-based carbonate. Letter m is the label of hydrogen or carbon atoms of [Et_4_N] cation. Number 1 is the label of hydrogen or carbon atoms of EG. Numbers 2, 3 and 4 are the labels of hydrogen or carbon atoms of EG-based carbonate.

In order to confirm our assumption, the ^1^H-^13^C 2D heteronuclear multiple bond correlation (HMBC) spectroscopy was used to further investigate the interaction between CO_2_ and [Et_4_N] [Car]:EG (1:2). The ^1^H-^13^C HMBC spectrum of [Et_4_N] [Car]:EG (1:2) after CO_2_ uptake was shown in Figure 3. As can be seen in Figure 3, H-2 correlated with C-3, and H-3 correlated not only with C-2 but also with C-4. The HMBC results confirmed that CO_2_ reacted with EG. On the basis of the HMBC results and previous results [23,24], the new peak at 157.7 (C-4) ppm was the carbonyl carbon of the carbonate formed by CO_2_ and EG. The new hydrogen peaks (H-2 and H-3) and the new carbon peaks (C-2 and C-3) can be ascribed to the methylene groups of the EG-based carbonate. Moreover, C-j did not correlate with H-2 or H-3, suggesting the peak at 160.3 (C-j) ppm was the carbonyl carbon of the Car-based carbonate [25]. The similar new peaks can also be found in the ^1^H and ^13^C NMR spectra of [Et_4_N] [Thy]:EG (1:2) after absorption (Figure S3).

**Figure 4:**
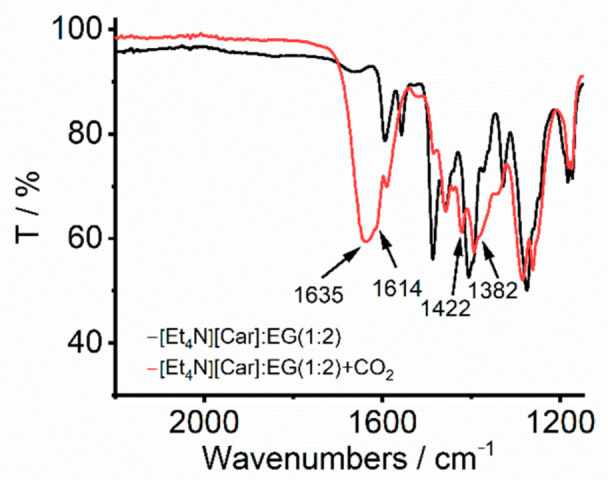
The FTIR spectra of [Et_4_N] [Car]:EG (1:2) before and after CO_2_ uptake.

The interaction between CO_2_ and [Et_4_N] [Car]:EG (1:2) was also supported by the FTIR results. As shown in Figure 4, compared to the FTIR spectrum of the virgin [Et_4_N] [Car]:EG (1:2), a new peak appeared near at 1635 cm^−1^, and a shoulder was observed at around 1614 cm^−1^. The peak at 1635 cm^−1^ can be attributed to the C=O stretching band of the EG-based carbonate, [24,26] whereas the shoulder at 1614 cm^−1^ was the C=O stretching band of the Car-based carbonate [27]. The FTIR results suggested again that CO_2_ reacted with both EG and the [Car]^−^ anion in the [Et_4_N] [Car]:EG (1:2). Moreover, after CO_2_ capture, the new band at 1422 cm^−1^ was assigned as the symmetric stretching band of COO^−^, and a shoulder near 1382 cm^−1^ was probably related to the doubly degenerate stretching of carbonate [28]. The peak at 1594 cm^−1^ attributed to aromatic ring mode shifted to 1591 cm^−1^ after CO_2_ absorption. Similarly, the C=O stretching modes of the EG-based and Thy-based carbonates appeared at 1627 and 1612 cm^−1^ (Figure S4), respectively.

**Scheme 2:**
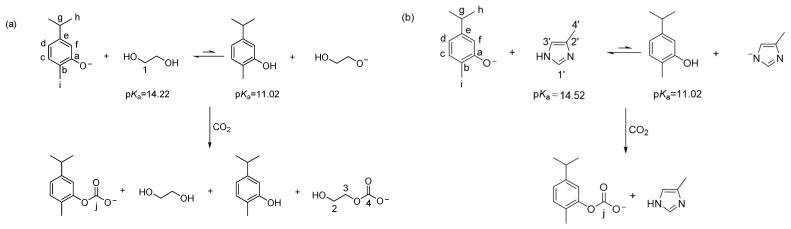
The possible reaction mechanisms of CO_2_ with [Et_4_N] [Car]:EG (1:2) (**a**) (the reaction between [Et_4_N] [Car]:EG (1:2) and CO_2_.) and [Et_4_N] [Car]:4CH_3_-Im (1:2) (**b**) (the reaction between [Et_4_N] [Car]:4CH_3_-Im (1:2) and CO_2_.). Letters from a to i are the labels of hydrogen or carbon atoms of [Car] anion. Letter j is the label of carbonyl carbon of Car-based carbonate. Number 1 is the label of hydrogen or carbon atom of EG. Numbers 2, 3 and 4 are the labels of hydrogen or carbon atoms of EG-based carbonate. Numbers 1′, 2′, 3′ and 4′ are the labels of hydrogen or carbon atoms of 4CH_3_-Im.

On the basis of the products and previous reports, a possible reaction mechanism between CO_2_ and [Et_4_N] [Car]:EG (1:2) can be proposed, which involved two steps (Scheme 2a). In the first step, there was an acid–base reaction between the anion [Car]^−^ and EG, producing carvacrol and the anion HO-CH_2_-CH_2_-O^−^. The position of equilibrium for this acid–base reaction can be determined by comparing the p*K*_a_ values of carvacrol (p*K*_a_ = 11.02) [29] and EG (p*K*_a_ = 14.22) [30], and the value of the equilibrium constant (*K*_eq_) can be obtained using the equation below (Equations (1) and (2)):

(1) $$pK_{\mathrm{eq}}=pK_{a}(\mathrm{EG})-pK_{a}(\mathrm{carvacrol})=3.20$$

(2) $$K_{\mathrm{eq}}{=10}^{-3.20}=6.31\times 10^{-4}$$

In the second step, CO_2_ reacted with the anions HO-CH_2_-CH_2_-O^−^ and [Car]^−^, forming the carbonated species.

Next, the absorption mechanism by the [Et_4_N] [Car]:4CH_3_-Im (1:2) solvent will be discussed. Similar to the [Et_4_N] [Car]:EG (1:2) system, an acid–base reaction also existed in the [Et_4_N] [Car]:4CH_3_-Im (1:2) solvent, in which the anion [Car]^−^ reacted with 4CH_3_-Im by forming carvacrol and the anion [4CH_3_-Im]^−^. The p*K*_eq_ value of this reaction can also be calculated using the p*K*_a_ values of carvacrol and 4CH_3_-Im (p*K*_a_ ≈ 14.52) [31]. Therefore, it is reasonable to assume that CO_2_ will react with the anions [Car]^−^ and [4CH_3_-Im]^−^ in the solvent, generating a carbonate species [-OCO_2_]^−^ and a carbamate species [N-OCO_2_]^−^, respectively. However, the NMR and FTIR results of [Et_4_N] [Car]:4CH_3_-Im (1:2) before and after CO_2_ absorption suggested that CO_2_ only reacted with the anion [Car]^−^ and did not react with the anion [4CH_3_-Im]^−^ (Scheme 2b). The details of the results are shown in the following sections.

**Figure 5:**
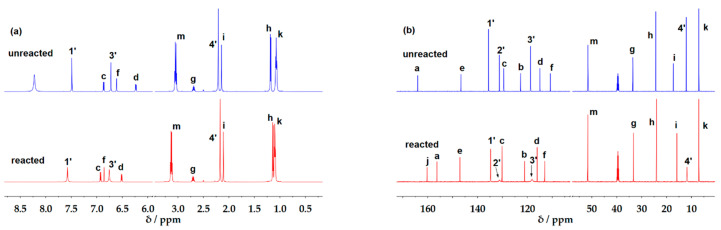
The ^1^H (**a**) and ^13^C (**b**) NMR spectra of [Et_4_N] [Car]:4CH_3_-Im (1:2) before and after CO_2_ uptake. Letters from a to i are the labels of hydrogen or carbon atoms of [Car] anion with and without CO_2_. Letters m and k are the labels of hydrogen or carbon atoms of [Et_4_N] cation. Letter j is the label of carbonyl carbon of Car-based carbonate. Numbers 1′, 2′, 3′ and 4′ are the labels of hydrogen or carbon atoms of 4CH_3_-Im.

**Figure 6:**
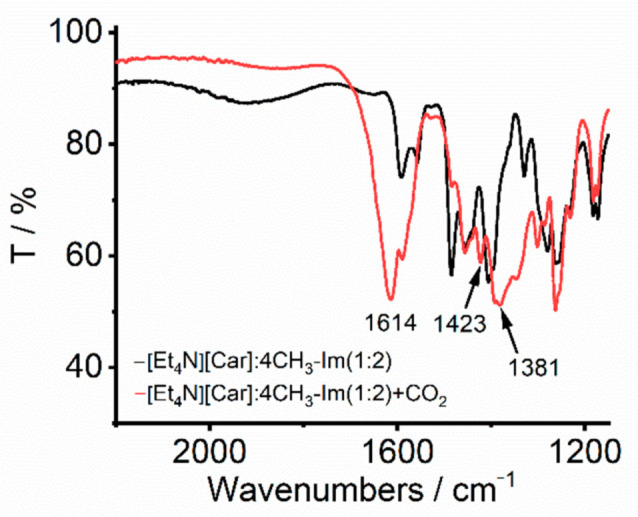
The FTIR spectra of [Et_4_N] [Car]:4CH_3_-Im (1:2) before and after CO_2_ uptake.

The NMR and FTIR spectra of [Et_4_N] [Car]:4CH_3_-Im (1:2) before and after CO_2_ absorption were presented in Figure 5 and Figure 6, respectively. As shown in Figure 5a, no new peaks can be found after absorption. The H-f and H-d obviously shifted downfield from 6.61 and 6.23 ppm to 6.86 and 6.50 ppm, respectively. However, the chemical shifts of the peaks of H-1′ (from 7.50 to 7.57 ppm) and H-3′ (from 6.72 to 6.75 ppm) on the imidazole ring changed very little. In the ^13^ C NMR spectra, only one new peak at 160.3 ppm (C-j) can be found after reaction, and this new carbon peak was the same as the Car-based carbonate peak (C-j) of the [Et_4_N] [Car]:EG (1:2) + CO_2_. Moreover, the C-a moved upfield obviously from 164.0 to 156.3 ppm, whereas C-f moved downfield from 110.8 to 113.0 ppm. The peak of C-1′ of 4CH_3_-Im shifted little from 135.6 to 134.8 ppm, and the chemical shift of the peak of C-2′ (131.2 ppm) did not move. These NMR results discussed above indicated that CO_2_ may only react with the anion [Car]^−^ in the DESs [Et_4_N] [Car]:4CH_3_-Im (1:2), and the reaction between CO_2_ and the anion [4CH_3_-Im]^−^ did not happen. Furthermore, the ^1^H and ^13^C NMR results (Figure S5) of [Et_4_N] [Thy]:4CH_3_-Im (1:2) with and without CO_2_ also showed the similar phenomena that CO_2_ reacted with the anion [Thy]^−^ rather than with [4CH_3_-Im]^−^.

As seen in Figure 6, a new, sharp peak at 1614 cm^−1^ attributed to C=O stretching band can be observed in the presence of CO_2_, which is in agreement with the C=O stretching frequency around at 1614 cm^−1^ of the Car-based carbonate in the [Et_4_N] [Car]:EG (1:2) + CO_2_ system, suggesting strongly that CO_2_ reacted with the anion [Car]^−^. Moreover, one new peak at 1423 cm^−1^ due to the symmetric stretching band of COO^−^ can be found. One new band at 1381 cm^−1^, probably related to the doubly degenerate stretching of carbonate, can also be observed. The peak at 1592 cm^−1^ attributed to aromatic ring mode shifted to 1590 cm^−1^ after CO_2_ absorption. For the [Et_4_N] [Thy]:4CH_3_-Im (1:2) solvent, one new peak at 1611 cm^−1^ corresponding to the C=O stretching of the carbonate species was also found in the FTIR spectra (Figure S6) after CO_2_ was loaded.

**Figure 7:**
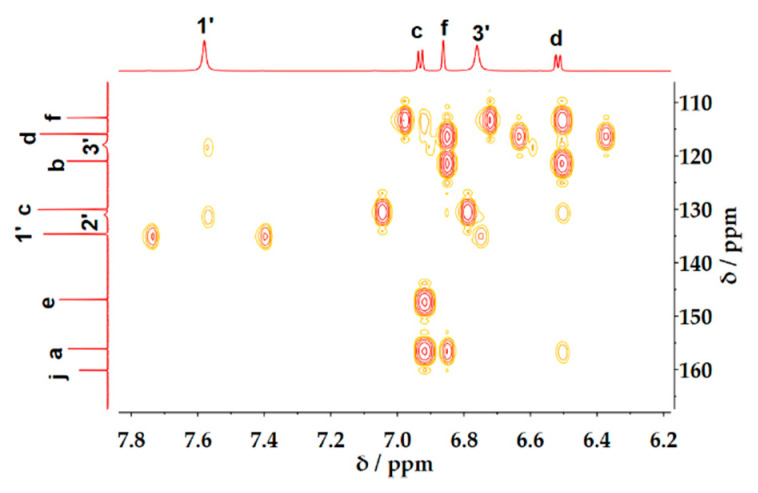
The ^1^H–^13^C HMBC spectrum of [Et_4_N] [Car]:4CH_3_-Im (1:2) + CO_2_. Letters from a to f are the labels of hydrogen or carbon atoms of Car-based carbonate. Letter j is the label of carbonyl carbon of Car-based carbonate. Numbers 1’, 2’ and 3’ are the labels of hydrogen or carbon atoms of 4CH_3_-Im.

**Figure 8:**
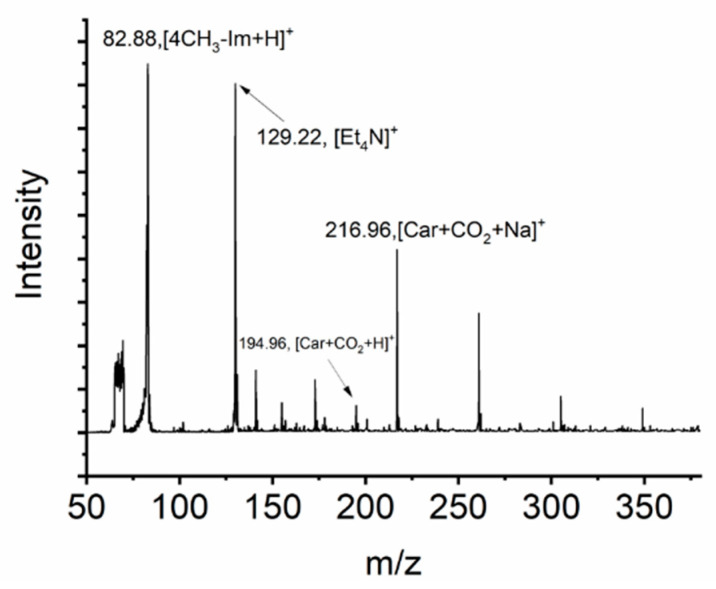
The electrospray ionization mass spectrum (ESI-MS) of CO_2_ loaded [Et_4_N] [Car]:4CH_3_-Im (1:2) solvent.

The reaction between CO_2_ and [Et_4_N] [Car]:4CH_3_-Im (1:2) was also studied using ^1^H-^13^C HMBC spectra and mass spectroscopy. In the HMBC spectra (Figure 7), there was no correlation peak between carbonyl carbon C-j and the H-1′ or H-3′on the imidazole ring, suggesting that CO_2_ was not bonded to the anion [4CH_3_-Im]^−^. In the mass spectrum (Figure 8), one peak at *m*/*z* 194.96 and one strong peak at *m*/*z* 216.96 can be found, corresponding to the ions [Car+CO_2_+H]^+^ and [Car+CO_2_+Na]^+^, respectively. In addition, no peaks related to [4CH_3_-Im+CO_2_+H]^+^ (*m*/*z* 127.1) or [4CH_3_-Im+CO_2_+Na]^+^ (*m*/*z* 149.1) can be observed. The HMBC and mass spectroscopy results confirmed again that CO_2_ reacted with [Car]^-^ and did not react with the anion [4CH_3_-Im]^-^ when it was captured by the DESs [Et_4_N] [Car]:4CH_3_-Im (1:2). The reaction mechanism between CO_2_ and [Et_4_N] [Car]:4CH_3_-Im (1:2) was proposed in Scheme 2b, which was different from that of [Et_4_N] [Car]:EG (1:2). The reason why CO_2_ did not react with the [4CH_3_-Im]^−^ may be because the steric hinderance of [N]^−^ of [4CH_3_-Im]^−^ was higher than that of [O]^−^ of [Car]^−^, leading CO_2_ to attack the anion [Car]^−^ rather than [4CH_3_-Im]^−^. For the [Et_4_N] [Car]:EG (1:2) system, the steric hinderance of [O]^−^ of [Car]^−^ was nearly the same to that of [O]^−^ of HO-CH_2_-CH_2_-O^−^, so CO_2_ reacted with both [Car]^−^ and HO-CH_2_-CH_2_-O^−^.

## 3. Materials and Methods

### 3.1. Materials and Characterizations

Tetraethylammonium hydroxide (35% *w*/*w*) aqueous solution was purchased from Alfa Aesar (Shanghai, China). 4-Methylimidazole was purchased TCI (Shanghai, China). Ethylene glycol (99.5%) was purchased J&K Scientific Ltd (Beijing, China). Thymol (98%) and carvacrol (99%) were purchased from Innochem (Beijing China). CO_2_ (99.995%) and N_2_ (≥99.99%) were obtained from Beijing ZG Special Gases Sci. and Tech. Co. Ltd. (Beijing, China). FTIR spectra were recorded on a Perkin-Elmer frontier spectrometer (PerkinElmer Corp., Waltham, MA, USA) with an attenuated total reflection (ATR) accessory (650 to 4000 cm^−1^). ^1^H NMR (600 MHz) and ^13^C NMR (151 MHz) spectra were obtained on a Bruker spectrometer (Bruker Biospin, Karlsruhe, Germany) and DMSO-d_6_ was used as the internal reference. Electrospray ionization mass spectra (ESI-MS) was recorded on a Thermo Scientific Quantum Ultra mass spectrometer (Thermo Scientific, Waltham, MA, USA). The viscosity was determined in an Anton Paar series MCR92 rheometer (Anton Paar, Graz, Austria). The melting point of DESs was obtained using a TA Instruments Q200 differential scanning calorimeter (New Castle, DE, USA) from −80 to 25 °C at a heating rate of 10 °C/min under a N_2_ atmosphere. Thermo-gravimetric analysis (TGA) was performed using a Mettler Toledo TG 3+ (Mettler Toledo, Greifensee, Switzerland) instrument from 25~400 °C at a heating rate of 10 °C/min under N_2_ atmosphere.

### 3.2. Synthesis of the ILs and DESs

At first, tetraethylammonium hydroxide ([Et_4_N] [OH]) (35% *w*/*w*) aqueous solution was mixed with phenol (thymol or carvacrol) at equimolar ratio ([Et_4_N] [OH]:phenol = 1:1) at room temperature, and then the solution was stirred about 2 h. After that, the water in the solution was removed by using a rotary evaporator, and the obtained IL was dried under vacuum at 80 °C prior to use.

The DES was prepared by stirring the mixture of IL ([Et_4_N] [Thy] or [Et_4_N] [Car]) with HBD (EG or 4CH_3_-Im) at desired molar ratio at 70 °C for about 1 h until a homogeneous liquid was formed.

NMR and FTIR data of DESs:

[Et_4_N][Car]:EG(1:2): ^1^H-NMR (600 MHz, DMSO-*d*_6_): δ = 6.71 (d, *J* = 7.38 Hz, 1H), 6.24 (s, 1H), 6.02 (d, *J* = 7.38 Hz, 1H), 3.49 (s, 8H), 3.05 (q, *J* = 7.32 Hz, 8H), 2.59–2.64 (m, 1H), 1.98 (s, 3H), 1.14 (d, *J* = 6.96 Hz, 6H), 1.07 (t, *J* = 7.32 Hz, 12H) ppm. ^13^C-NMR (151 MHz, DMSO-*d*_6_): δ = 167.3, 146.7, 129.4, 123.5, 116.2, 108.8, 63.8, 51.8, 33.9, 24.7, 17.7, 7.3 ppm. FTIR: ν = 3204, 2925, 2867, 1594, 1557, 1487, 1458, 1406, 1328, 1275, 1183, 1173, 1092, 1047, 990, 944, 869, 786, 700, 661 cm^−1^.

[Et_4_N][Car]:4CH_3_-Im(1:2): ^1^H-NMR (600 MHz, DMSO-*d*_6_): δ = 7.49 (s, 2H), 6.86 (d, *J* = 7.44 Hz, 1H), 6.72 (s, 2H), 6.61 (s, 1H), 6.23 (d, *J* = 7.44 Hz, 1H), 3.04 (q, *J* = 7.32 Hz, 8H), 2.67–2.71 (m, 1H), 2.21 (s, 6H), 2.15 (s, 3H), 1.18 (d, *J* = 6.96 Hz, 6H), 1.07 (t, *J* = 7.32 Hz, 12H) ppm. ^13^C-NMR (151 MHz, DMSO-*d*_6_): δ = 164.0, 146.7, 135.6, 131.2, 129.5, 122.7, 118.7, 114.9, 110.8, 51.6, 33.5, 24.3, 17.3, 12.1, 7.0 ppm. FTIR: ν = 2957, 2866, 1592, 1558, 1485, 1456, 1406, 1329, 1279, 1263, 1182, 1172, 1104, 989, 921, 869, 786, 665 cm^−1^.

[Et_4_N][Thy]:EG(1:2): ^1^H-NMR (600 MHz, DMSO-*d*_6_): δ = 6.76 (d, *J* = 7.50 Hz, 1H), 6.33 (s, 1H), 6.09 (d, *J* = 7.50 Hz, 1H), 3.49 (s, 8H), 3.27–3.34 (m, 1H), 3.06 (q, *J* = 7.32 Hz, 8H), 2.10 (s, 3H), 1.08–1.11 (m, 18H) ppm. ^13^C-NMR (151 MHz, DMSO-*d*_6_): δ = 164.4, 134.2, 133.3, 124.3, 118.7, 112.7, 63.6, 51.6, 25.7, 23.4, 21.2, 7.0 ppm. FTIR: ν = 2921, 2863, 1594, 1488, 1455, 1396, 1340, 1290, 1269, 1166, 1090, 1047, 1001, 951, 858, 785, 740 cm^−1^.

[Et_4_N][Thy]:4CH_3_-Im(1:2): ^1^H-NMR (600 MHz, DMSO-*d*_6_): δ = 7.50 (s, 2H), 6.89 (d, *J* = 7.56 Hz, 1H), 6.73 (s, 2H), 6.61 (s, 1H), 6.26 (d, *J* = 7.56 Hz, 1H), 3.41–3.45 (m, 1H), 3.05 (q, *J* = 7.32 Hz, 8H), 2.21 (s, 6H), 2.15 (s, 3H), 1.20 (d, *J* = 7.02 Hz, 6H), 1.08 (t, *J* = 7.32 Hz, 12H) ppm. ^13^C-NMR (151 MHz, DMSO-*d*_6_): δ = 162.1, 135.3, 134.5, 132.9, 131.2, 124.6, 118.6, 117.9, 114.3, 51.6, 26.0, 23.2, 21.1, 12.0, 7.0 ppm. FTIR: ν = 2954, 2864, 1592, 1484, 1450, 1395, 1339, 1291, 1263, 1149, 1105, 999, 785, 740, 667 cm^−1^.

### 3.3. Absorption and Desorption of CO_2_

DES (about 2.0 g) was charged into a glass tube with an inner diameter of 10 mm, and the glass tube was immersed in a water bath at 25 °C. CO_2_ was bubbled through the solvent in the tube at a flow rate of 50 mL/min. The weight of the tube during absorption process was determined by an electronic balance with an accuracy of ±0.1 mg. The CO_2_ absorbed by the DES can be calculated through the weight change of the tube with and without CO_2_.

In the desorption process, the glass tube was immersed in an oil bath at 80 °C. N_2_ was bubbled through the solvent in the tube at a flow rate of 40 mL/min.

## 4. Conclusions

The four anion-functionalized DESs based on bio-derived phenols could efficiently capture CO_2_, up to 0.90 mol CO_2_/mol solvent. Hydrogen bond donors EG and 4CH_3_-Im impact greatly on the absorption mechanism by these DESs, and the reaction pathway between CO_2_ and absorbents can be tuned by changing HBDs. CO_2_ reacted with both EG and anions in the solvent when EG acted as the HBDs, forming the EG-based carbonate species and phenol-based carbonate species. Nevertheless, when 4CH_3_-Im was used as the HBDs, CO_2_ only reacted with the phenolic anion in the solvent. The different reaction pathway may be due to the steric hinderance differences of the functional groups of the HBDs. We think the findings of this work will be very useful to the design of new, efficient anion-functionalized DESs for carbon capture and utilizations, which will promote the development of the DESs community.

## Data Availability

Not applicable.

## Supplementary Materials

The following are available online, Table S1: The melting point (*T*_m_), decomposition temperature (*T*_d_) and viscosity of DESs, Table S2: Comparison of CO_2_ capacities in this study with previously reported DESs, Figure S1: The impact of temperature on the CO_2_ absorption by DESs [Et_4_N] [Car]:EG (1:2) and [Et_4_N][Car]:4CH_3_-Im (1:2), Figure S2: The five consecutive CO_2_ absorption–desorption cycles of [Et_4_N][Car]:EG(1:2), Figure S3: The ^1^H (a) and ^13^C (b) NMR spectra of [Et_4_N] [Thy]:EG (1:2) before and after CO_2_ uptake, Figure S4: The FTIR spectra of [Et_4_N] [Thy]:EG (1:2) before and after CO_2_ uptake, Figure S5: The ^1^H (a) and ^13^C (b) NMR spectra of [Et_4_N] [Thy]:4CH_3_-Im (1:2) before and after CO_2_ uptake, Figure S6: The FTIR spectra of [Et_4_N] [Thy]:4CH_3_-Im (1:2) before and after CO_2_ uptake.

## Sample Availability

Samples of the compounds used in this study are available from the authors.
