# Supplementary material for: The Influence of Hydrogen Bond Donors on the CO2 Absorption Mechanism by the Bio-Phenol-Based Deep Eutectic Solvents
Source: Molecules. 2021 Nov 26;26(23):7167. doi: 10.3390/molecules26237167 (PMC8658771; doi:10.3390/molecules26237167)
Supplement: Supplementary file 1 [file molecules-26-07167-s001.zip › molecules-1441485-supplementary.pdf]

Supplementary Materials

The Influence of Hydrogen Bond Donors on the CO_2 Absorption Mechanism by the Bio-Phenol-Based Deep Eutectic Solvents

Ze Wang, Zonghua Wang, Jie Chen, Congyi Wu and Dezhong Yang *

School of Science, China University of Geosciences, Beijing, 100083, China; wangze666wz @163.com (Z.W.); wzh13263359399@126.com (Z.W.); chenjie@cugb.edu.cn (J.C.); wucongyi@cugb.edu.cn (C.W.)

* Correspondence: yangdz@cugb.edu.cn.

Table S1. The melting point (T_m), decomposition temperature (T_d) and viscosity of DESs.

DESs	T_m / °C	T_d / °C	η/ mPa • s (25 °C)
$[Et_4N][Car]:EG(1:2)$	Not observed	100	279
$[Et_4N][Car]:4CH_3\text{-}Im(1:2)$	-47	104	891
$[Et_4N][Thy]:EG(1:2)$	Not observed	101	292
$[Et_4N][Thy]:4CH_3\text{-}Im(1:2)$	-45	115	1298

Table S2. Comparison of CO_2 capacities in this study with previously reported DESs.

Absorbents	T/ °C	P/bar	Capacity (mol CO_2/mol solvent)	References
$[Et_4N][Thy]:EG$ (1:2)	25	1.0	0.90	This work
$[Et_4N][Thy]:4CH_3\text{-}Im$ (1:2)	25	1.0	0.90	This work
$[Et_4N][Car]:4CH_3\text{-}Im$ (1:2)	25	1.0	0.88	This work
$[Et_4N][Car]:EG$ (1:2)	25	1.0	0.87	This work
[TETA]Cl-EG (1:3)	30	1.0	1.521	[1]
DBN−BmimCl−Im (1:1:1)	25	1.0	1.02	[2]
DBN−BmimCl−Im (1:1:2)	25	1.0	0.97	[2]
$[Et_4N][Im]$–EG (1:2)	25	1.0	0.94	[3]
$[Et_4N][Triz]$–EG (1:2)	25	1.0	0.92	[3]
$[Et_4P][Im]$–EG (1:2)	25	1.0	0.91	[3]
$[Et_4P][Triz]$–EG (1:2)	25	1.0	0.91	[3]
[DBUH][MLU]-EG (1:1)	40	1.0	0.90	[4]
[EMIM][2-Npyr]-EG (1:2)	25	1.0	0.85	[5]
[MEAH][Im]:EG (1:1)	25	1.0	0.62	[6]
L-arginine: glycerol (1:5)	80	1.0	0.403	[7]
L-arginine: glycerol (1:6)	80	1.0	0.457	[7]
MEA:BmimCl (1:1)	25	1.0	0.45	[8]
DBN-DMLU (2:1)	45	1.0	0.36	[9]

MEA: monoethanolamine; [TETA]Cl: triethylenetetramine and HCl mixture; DBN and DBU are superbases; DBUH: protonated DBU; MEAH: protonated MEA; BmimCl: 1-butyl-3-methylimidazolium chloride; Im: imidazole; [Im]: imidazolate; [Triz]: triazolate; $[Et_4P]$: tetraethylphosphonium; [MLU]: deprotonated methyl urea; [EMIM][2-Npyr]: 1-ethyl-3-methylimidazolium 2-cyanopyrrolide; DMLU: dimethylolurea.

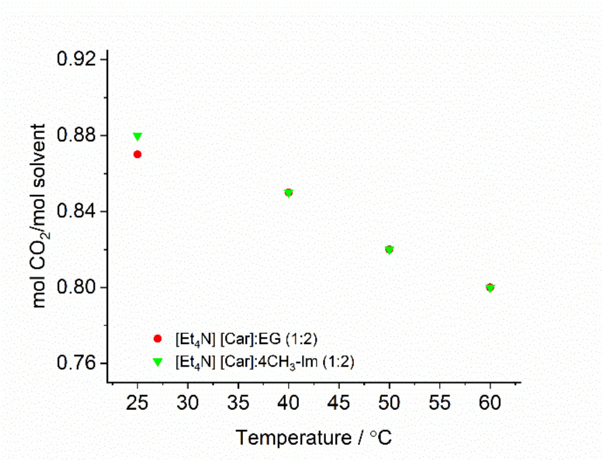

Figure S1. The impact of temperature on the CO_2 absorption by DESs [Et_4N] [Car]:EG (1:2) and [Et_4N][Car]:4CH_3-Im (1:2).

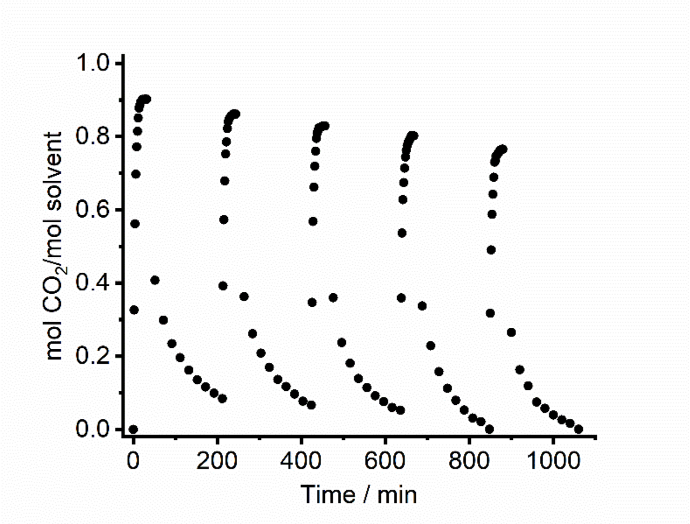

Figure S2. The five consecutive CO_2 absorption–desorption cycles of [Et_4N][Car]:EG(1:2).

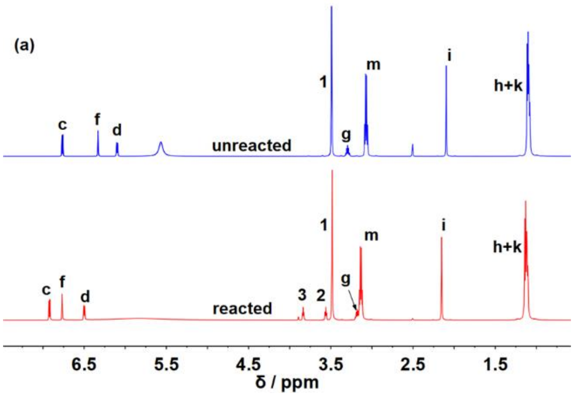

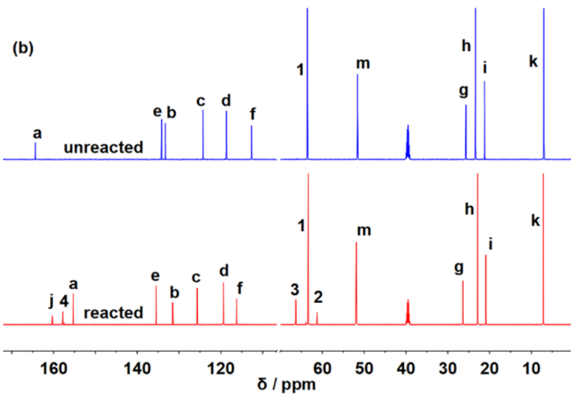

Figure S3. The ^{1}H (a) and ^{13}C (b) NMR spectra of $[Et_4N]$ [Thy]:EG (1:2) before and after CO_2 uptake. Letters m and k are the labels of hydrogen or carbon atoms of $[Et_4N]$ cation. Letter j is the label of carbonyl carbon of Car-based carbonate. Numbers 1 is the label of hydrogen or carbon atom of EG. Numbers 2, 3 and 4 are the labels of hydrogen or carbon atoms of EG-based carbonate.

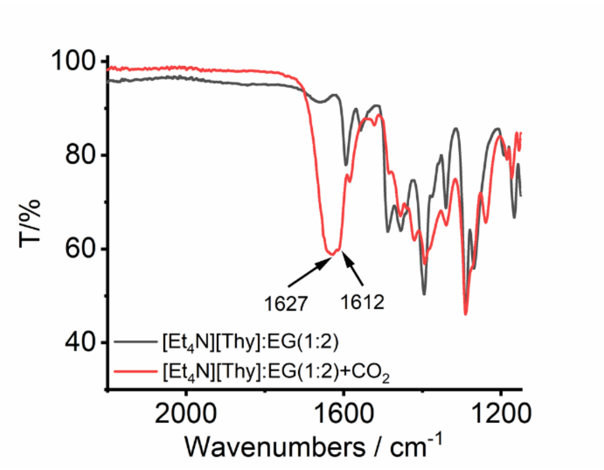

Figure S4. The FTIR spectra of $[Et_4N]$ [Thy]:EG (1:2) before and after CO_2 uptake.

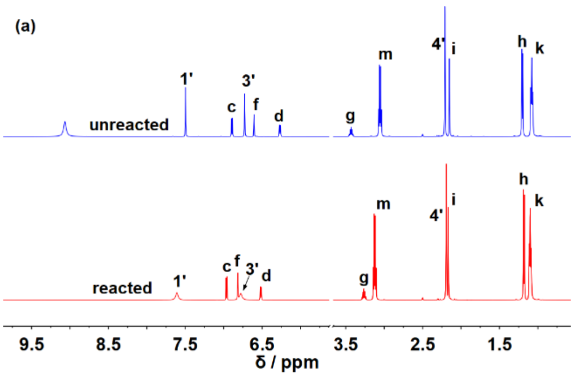

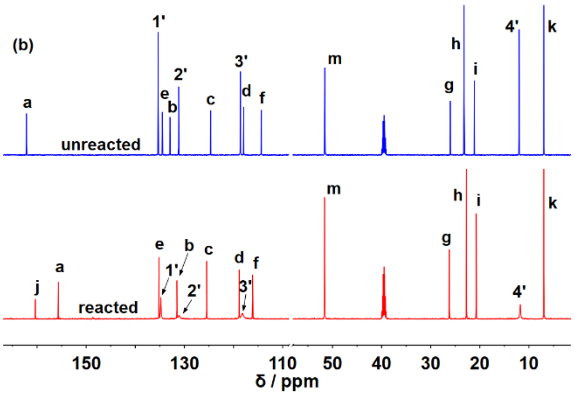

Figure S5. The ^{1}H (a) and ^{13}C (b) NMR spectra of [Et4N] [Thy]:4CH3-Im (1:2) before and after CO_2 uptake. Letters from a to i are the labels of hydrogen or carbon atoms of [Car] anion with and without CO_2. Letters m and k are the labels of hydrogen or carbon atoms of [Et4N] cation. Letter j is the label of carbonyl carbon of Car-based carbonate. Numbers 1′, 2′, 3′ and 4′ are the labels of hydrogen or carbon atoms of 4CH3-Im.

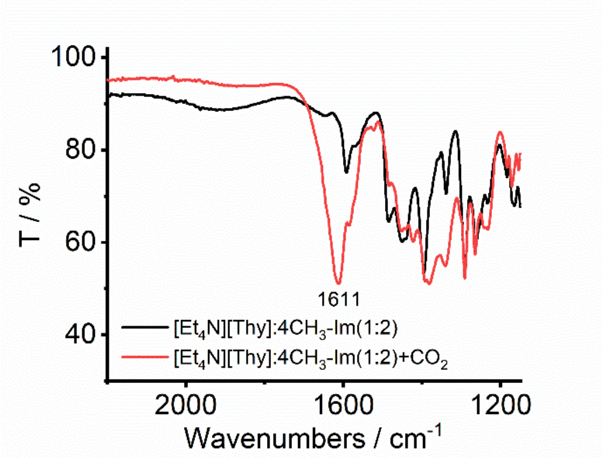

Figure S6. The FTIR spectra of [Et4N] [Thy]:4CH3-Im (1:2) before and after CO_2 uptake.

References

1. Zhang, K.; Hou, Y.; Wang, Y.; Wang, K.; Ren, S.; Wu, W., Efficient and Reversible Absorption of CO2 by Functional Deep Eutectic Solvents. *Energy Fuels* **2018,** 32, (7), 7727-7733.
2. Zhang, N.; Huang, Z.; Zhang, H.; Ma, J.; Jiang, B.; Zhang, L., Highly Efficient and Reversible CO2 Capture by Task-Specific Deep Eutectic Solvents. *Ind. Eng. Chem. Res.* **2019,** 58, (29), 13321-13329.
3. 3. Cui, G.; Lv, M.; Yang, D., Efficient CO2 absorption by azolide-based deep eutectic solvents. *Chem. Commun.* **2019,** 55, (10), 1426-1429.
4. 4. Fu, H.; Wang, X.; Sang, H.; Liu, J.; Lin, X.; Zhang, L., Highly efficient absorption of carbon dioxide by EG-assisted DBU-based deep eutectic solvents. *J. CO_2 Util.* **2021,** 43, 101372.
5. 5. Lee, Y.-Y.; Penley, D.; Klemm, A.; Dean, W.; Gurkan, B., Deep Eutectic Solvent Formed by Imidazolium Cyanopyrrolide and Ethylene Glycol for Reactive CO2 Separations. *ACS Sustainable Chem. Eng.* **2021,** 9, (3), 1090-1098.
6. 6. Mukesh, C.; Khokarale, S. G.; Virtanen, P.; Mikkola, J.-P., Rapid desorption of CO2 from deep eutectic solvents based on polyamines at lower temperatures: an alternative technology with industrial potential. *Sustainable Energy Fuels* **2019,** 3, (8), 2125-2134.
7. 7. Ren, H.; Lian, S.; Wang, X.; Zhang, Y.; Duan, E., Exploiting the hydrophilic role of natural deep eutectic solvents for greening CO2 capture. *J. Cleaner Prod.* **2018,** 193, 802-810.
8. 8. Cao, L.; Huang, J.; Zhang, X.; Zhang, S.; Gao, J.; Zeng, S., Imidazole tailored deep eutectic solvents for CO2 capture enhanced by hydrogen bonds. *Phys. Chem. Chem. Phys.* **2015,** 17, (41), 27306-27316.
9. Jiang, B.; Ma, J.; Yang, N.; Huang, Z.; Zhang, N.; Tantai, X.; Sun, Y.; Zhang, L., Superbase/Acylamido-Based Deep Eutectic Solvents for Multiple-Site Efficient CO2 Absorption. *Energy Fuels* **2019,** 33, (8), 7569-7577.
